# An unusual case of cardiac dysfunction after left ventricular reconstruction

**DOI:** 10.1186/1749-8090-1-28

**Published:** 2006-09-19

**Authors:** Lynn M Fedoruk, Irving L Kron

**Affiliations:** 1 From the Division of Thoracic and Cardiovascular Surgery, Department of Surgery, University of Virginia Health System, Charlottesville, Virginia, USA

## Abstract

This report describes an unusual cause of low cardiac output after coronary artery bypass grafting and left ventricular remodeling. It details left ventricular remodeling techniques and discusses the most recent advances and outcomes. As well, significant attention is paid to the issues surrounding failure to separate from cardiopulmonary bypass.

## Case report

**Figure 1:**
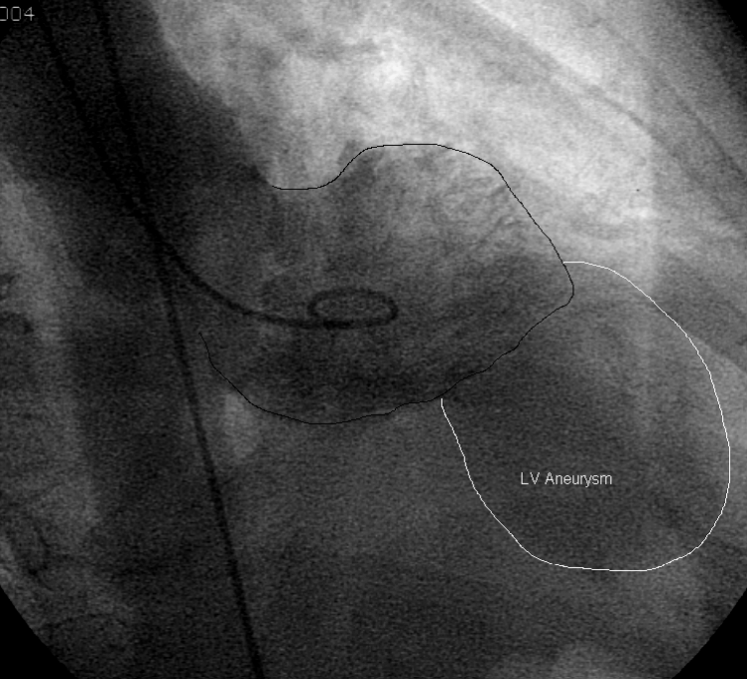
Left Ventriculogram demonstrating anterioapical left ventricular aneurysm.

A sixty-nine year old female with known three vessel coronary artery disease presented to the emergency department of a peripheral hospital with dyspnea and chest pain. Previous angiography demonstrated a 50% proximal left anterior descending (LAD) artery obstruction with a 99% occlusion to the mid and distal portions, a 50% proximal circumflex lesion and a 90% distal right coronary artery (RCA) obstruction with poor vessels distal to the occlusion. Subsequent to that angiogram, percutaneous interventions (PCI) were performed to all three vessels, with bare metal stents being placed in the circumflex and LAD arteries and percutaneous transluminal coronary angioplasty (PTCA) being performed on the RCA. A more recent angiogram performed four months prior to admission demonstrated an unsuccessful PTCA of the RCA and occlusion of the distal LAD. The circumflex vessel had minimal disease. A large anterioapical aneurysm was demonstrated by the ventriculogram performed at that time (Figure 1).

Upon arrival to the emergency department, she required intubation for the management of her pulmonary edema. A transthoracic echocardiogram demonstrated decreased left ventricular (LV) function with an ejection fraction estimated at thirty percent. An extensive 8 by 10 centimeter anterioapical aneurysm was visualized. No mitral regurgitation was present and thrombus was not seen within the ventricle or aneurysm. After stabilization, she was transferred to our institution for ongoing care and surgical consideration.

Part history was remarkable for severe COPD (FEV1 25% predicted), atrial fibrillation and ileus. After considerable workup, a decision was made to perform coronary artery bypass grafting to the LAD, a left ventricular aneurysm repair and an atrial ablation.

## Surgical technique

In the operating room, standard aortic and single three-stage venous cannula were placed. Care was taken to manipulate the heart as minimally as possible. Antegrade, cold blood (6–8°C) cardioplegia was utilized. After aortic cross clamping, the initial electromechanical arrest was readily achieved with 300 milliliters of cardioplegia. The total initial dose of cardioplegia was one liter. Subsequent dosing was performed every 15 – 20 minutes in an antegrade fashion via the aortic cardioplegia cannula.

At this point, the extensive adhesions around the aneurysm were sharply dissected away allowing for easier visualization of the left and right pulmonary veins. The pulmonary veins were ablated in a circumferential fashion by radiofrequency, ablating first the right superior and inferior pulmonary veins en bloc followed by the left.

**Figure 2:**
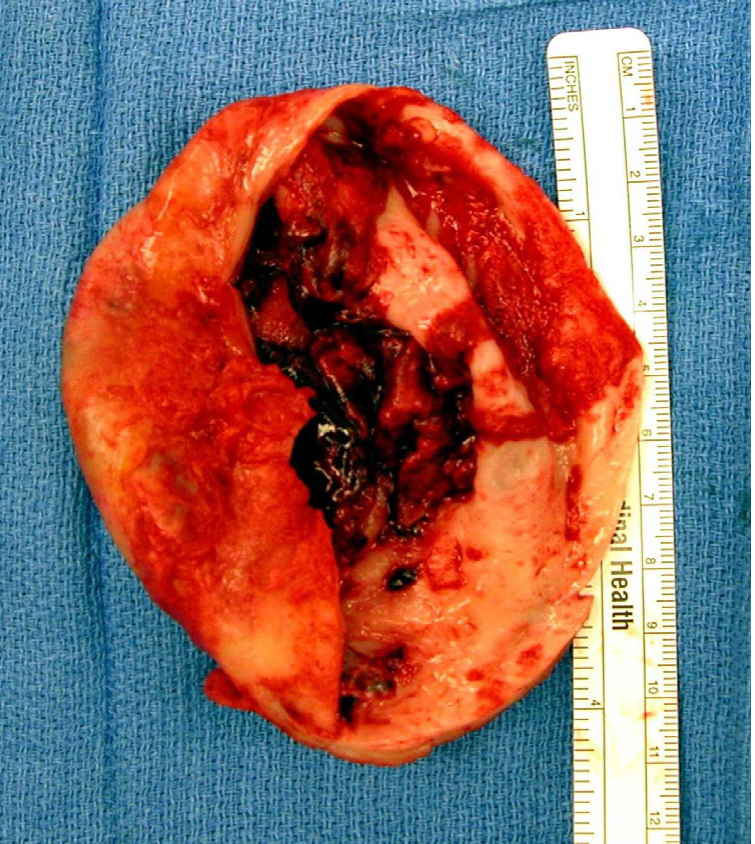
Photograph of resected 10 cm aneurysm. Note the intramural thrombus

The aneurysm was then opened in roughly its midline parallel to the LAD and three large clots, each measuring approximately 2–3 centimeters in diameter, were removed. After removal of a significant amount of the body of the aneurysm as delineated by the thin, scarred wall (Figure 2), the ventricle was examined and irrigated to ensure no further clots were present. The demarcation between the scar and viable myocardium was clearly visible and a 2-0 prolene suture (Ethicon, Summerville, NJ) was placed in a purse string manner around the transition point (Fontan stitch). This was then approximated leaving an oval opening approximately two centimeters by three centimeters. An endoventricular patch was then fashioned utilizing a dacron graft (DuPont company, Wilmington, DE) and this was sutured in place at the level of the pursestring suture with 2-0 prolene. The ventricle was then closed in two layers utilizing 3-0 prolene.

At this point, the LAD was examined and found to be diffusely diseased. An area free of palpable atherosclerosis was isolated and arteriotomy performed. Surprisingly, the vessel was of reasonable caliber and the left internal mammary artery was used to bypass this artery. After thorough de-airing and a 'hot shot' dose of cardioplegia, the cross clamp was removed. Total cross clamp time was 60 minutes initially.

After placing atrial and ventricular wires for electrical asystole, attempts were made to separate from cardiopulmonary bypass (CPB). Initial attempts to wean from CPB were unsuccessful. The patient was hypotensive despite multiple ionotropes (epinephrine, norepinephrine, milrinone and vasopressin). The cardiac index was 1.3 liters/minute/m^2 ^and the left ventricle was distended and diffusely hypokinetic by transesophageal echocardiogram (TEE). Minimal air was demonstrated. The ECG displayed a wide complex atrioventricular paced rhythm. CPB was resumed and an intra-aortic balloon pump (IABP) was placed. A second attempt to wean from CPB was made. Again, significant global LV dysfunction was observed. Repeat TEE demonstrated the possibility of clot in the aortic root around the area of the left main coronary ostia. CPB was resumed for a second time, the cross clamp was reapplied and cold blood cardioplegia reinstituted. Again, prompt arrest was achieved.

Upon opening the ascending aorta, a 0.5 centimeter by 1.0 centimeter clot was found adherent to the left main coronary ostium. This was easily removed and after inspection to ensure no clot had migrated down the left main coronary artery the aorta was closed with 3-0 prolene.

After removing the cross clamp the second time, spontaneous sinus rhythm resumed. The patient was then successfully weaned from CPB with the aid of the IABP as well as multiple ionotropes. Total cross clamp time was 70 minutes (60 + 10) and the total CPB time was 175 minutes (130 + 45 minutes).

The patient was taken to the post operative intensive care unit and the IABP was removed after 24 hours. All ionotropes were discontinued by post operative day three. After a prolonged postoperative course secondary to ileus, the patient was discharged home.

## Discussion

There are multiple well recognized indications for aneurysm resection including congestive heart failure (CHF), angina, ventricular arrhythmias and distal embolization from thrombus propagated in the aneurysm cavity [1]. Initial aneurysm resection procedures were performed using a linear closure technique. This technique has subsequently undergone multiple modifications including a circular purse string closure (Jatene) [2], an endoventricular circular patch plasty (Dor) [3], modified linear closure technique (Mickleborough) [4] and endoaneurysmorrhaphy (Cooley). The objective behind these newer ventricular reconstruction techniques is to restore ventricular size and shape to prevent progressive ventricular remodeling. This is thought to improve LV efficiency by decreasing paradoxical wall motion, decreasing myocardial wall tension and thus decreasing myocardial oxygen requirements and systolic shortening requirements for ejection. Ultimately, this improves ventricular function and decreases the degree of CHF.

One of the critical points to recognize about this case is that the global ventricular dysfunction that occurred during the attempt to wean from CPB is rarely seen with this type of surgery. Regardless of the type of remodeling approach used, early mortality associated with these procedures is low, ranging from 12% in the initial series by Dor [3] to 2.6 – 4% in the more recent reports published by Mickleborough [4] and the RESTORE group [5]. The immediate post operative studies demonstrated an increase in ejection fraction averaging 9 – 10% [4,5] and a decrease in left ventricular end systolic volume index of 24 ml/m^2 ^[5]. Further, the RESTORE group had an 8.2% requirement for IABP for low cardiac output syndrome (LCOS) and a 0.7% LVAD use [5]. In both groups, no intra-operative deaths were reported. It is important to recognize that despite poor preoperative ventricular function, remodeling procedures themselves are generally not associated with acute ventricular dysfunction leading to LCOS and failure to separate from CPB, especially in the setting of a normal mitral valve. As with any other patient who fails to separate from CPB, an aggressive search for the cause must be undertaken.

The reasons for failure to wean from CPB can be divided into two broad categories: general or patient/case specific. Although a complete list is too comprehensive for this case report, table 1 describes many issues associated with failure to wean from CPB. In general, the minimum investigations necessary to aid in ascertaining the cause behind the failure to separate from CPB include arterial blood gases, electrolyte studies (including potassium), electrocardiography, transesophageal echocardiography and central monitoring. These should give insight into the underlying cause and allow for immediate correction.

**Table 1:** Etiology and investigation of post CPB ventricular dysfunction

	**Cause**		**Investigation**	**Finding**
**General**	Exacerbation of preoperative ventricular dysfunction with relative intolerance to cardioplegic asystolic, hypoxic arrest		TEE	Global or regional wall motion abnormality
	Reperfusion injury		TEE	Global wall motion abnormality
	Inadequate myocardial protection (underlying coronary anatomy, route of cardioplegia, type of cardioplegia)		TEE	Global wall motion abnormality
**Case/Patient Specific**	Ischemia/infarction	Vessel spasm (native coronaries, internal mammary artery)	ECG, TEE, graft flow	ECG changes, regional wall motion abnormality, poor graft flow
		Emboli (air, clot, particulate matter)	ECG, TEE, graft flow	ECG changes, regional wall motion abnormality, poor graft flow
		Technical graft anastomotic tissues	ECG, TEE, graft flow	ECG changes, regional wall motion abnormality, poor graft flow
		Kink/clotting of bypass grafts, native vessels	ECG, TEE, graft flow, inspection	ECG changes, regional wall motion abnormality, poor graft flow
	Incomplete revascularization	Non graftable vessels		
		Known intrinsic disease		
	Metabolic	Hypoxia, Hypercarbia	ABG, electrolytes, check ventilation	
		Hypokalemia, hyperkalemia	electrolytes	
	Uncorrected pathology	Hypertrophic cardiomyopathy	TEE	Abnormal outflow gradient, SAM
		Valve gradients	TEE	Abnormal valve gradient
		Shunts	TEE	Abnormal Doppler jet
	Mechanical Issues	Prosthetic valve function	TEE	Poor leaflet motion, abnormal gradient
		Intracardiac shunt (ASD, VSD)	TEE	Abnormal Doppler jet
	Conduction Issues	Bradycardia	ECG	Heart rate less than 60
		Atrioventricular dissociation	ECG	3^rd ^degree heart block
		Atrial Fibrillation	ECG, ABG, electrolytes	Hypoxia, electrolyte abnormality
		Ventricular arrythmias	ECG, ABG, electrolytes	Hypoxia, electrolyte abnormality
	Pulmonary hypertension	Preexisting elevated pulmonary pressures, hypoxia, hypercarbia, fluid overload	Swan Ganz monitoring, ABG	Elevated Pulmonary artery pressures, hypoxia, hypercarbia, RV distention
	Right Ventricular Failure	Elevated pulmonary pressures, inadequate myocardial protection, emboli to native or bypass circulation, fluid overload	Swan Ganz monitoring, ABG, TEE	RV distention, poor RV wall motion, elevated pulmonary artery pressure, elevated central venous pressure

ABG = arterial blood gas, ECG = electrocardiogram, RV = right ventricle, SAM = systolic anterior motion of mitral valve leaflet, TEE = Tranesophageal echocardiogram

Retrospectively, the cause of this patient's decompensation was embolization to the left main coronary ostium at the outside hospital. We presume that the antegrade cardioplegia utilized during the operation further impacted the clot into the left main, causing global left ventricular ischemia and myocardial dysfunction. Once the cause was identified and corrected, myocardial dysfunction improved and the patient was able to separate from CPB.

## Declaration of interests

The author(s) declare that they have no competing interests.
